# Extracellular Vesicle as a Source of Alzheimer’s Biomarkers: Opportunities and Challenges

**DOI:** 10.3390/ijms20071728

**Published:** 2019-04-08

**Authors:** Seongju Lee, Sakulrat Mankhong, Ju-Hee Kang

**Affiliations:** 1 Department of Anatomy, College of Medicine, Inha University, Incheon 22212, Korea; lees@inha.ac.kr; 2 Hypoxia-related Disease Research Center, College of Medicine, Inha University, Incheon 22212, Korea; sakulratkulrat@gmail.com; 3 Department of Pharmacology, College of Medicine, Inha University, Incheon 22212, Korea

**Keywords:** Alzheimer’s disease, extracellular vesicles, biomarker, exosome, central nervous system, standardization

## Abstract

Alzheimer’s disease (AD) is a chronic progressive neurodegenerative disease characterized by memory decline and cognitive dysfunction. Although the primary causes of AD are not clear, it is widely accepted that the accumulation of amyloid beta (Aβ) and consecutive hyper-phosphorylation of tau, synaptic loss, oxidative stress and neuronal death might play a vital role in AD pathogenesis. Recently, it has been widely suggested that extracellular vesicles (EVs), which are released from virtually all cell types, are a mediator in regulating AD pathogenesis. Clinical evidence for the diagnostic performance of EV-associated biomarkers, particularly exosome biomarkers in the blood, is also emerging. In this review, we briefly introduce the biological function of EVs in the central nervous system and discuss the roles of EVs in AD pathogenesis. In particular, the roles of EVs associated with autophagy and lysosomal degradation systems in AD proteinopathy and in disease propagation are discussed. Next, we summarize candidates for biochemical AD biomarkers in EVs, including proteins and miRNAs. The accumulating data brings hope that the application of EVs will be helpful for early diagnostics and the identification of new therapeutic targets for AD. However, at the same time, there are several challenges in developing valid EV biomarkers. We highlight considerations for the development of AD biomarkers from circulating EVs, which includes the standardization of pre-analytical sources of variability, yield and purity of isolated EVs and quantification of EV biomarkers. The development of valid EV AD biomarkers may be facilitated by collaboration between investigators and the industry.

## 1. Extracellular Vesicles: An Overview 

### 1.1. Biogenesis and Secretion

Extracellular vesicles (EVs) are released from virtually all cell types, including cells in the central nervous system (CNS) such as oligodendrocytes, neurons, astrocytes, microglia and Schwann cells, as well as endothelial cells [1,2]. EVs can facilitate neuron-glia communication, promote neuronal progression and regeneration and also contribute to the development of glioblastoma and neurodegenerative diseases [3,4,5,6]. EVs are comprised of shedding exosomes (40 to 100 nm in diameter), microvesicles and apoptotic bodies, which vary in size, cellular origin, process biogenesis and biophysical properties [7]. Microvesicles and exosomes originate from healthy resting or stimulated cells, while the apoptotic bodies are shed from dying cells [8].

Currently, the most reliable machinery for the biogenesis of EVs is the endosomal sorting complexes required for transport (ESCRT) [9,10]. Microvesicles directly pinch off from the plasma membrane, which is likely to be a simple process. Conversely, these organizations are required for several energy-dependent mechanisms, including the asymmetric movement of the plasma membrane by aminophospholipid translocase [11], membrane curvature [12,13] and cytoskeleton rearrangement [14]. Whereas microvesicles are heterogeneous in size, exosomes that originate from multivesicular bodies (MVBs) of the endosomal system are relatively homogeneous in size and density. MVBs are endosomal organelles containing intraluminal vesicles (ILVs) within a membrane-enclosed lumen. MVBs can fuse with the plasma membrane, leading to the release of the ILVs as exosomes into the extracellular space [15,16]. The endosomal sorting complexes required for transport (ESCRT) machinery comprises ESCRT-0, -I, -II and -III along with accessory proteins (e.g., Alix, VPS4 and VTA-1) which consecutively bind future exosomes with cargoes and form ILVs incorporating those cargoes [9,17,18]. An alternative pathway of exosomal formation can be mediated by sphingolipid ceramide and tetraspanins [19,20,21]. However, this pathway may not be entirely independent but could compensate to allow ILV formation without ESCRT. Upon formation, microvesicles may be a direct consequence of their generation and fission. On the contrary, the release of exosomes necessitates additional steps to sort cargoes to ILVs in MVBs, subsequently fusing MVBs with the plasma membrane [22]. MVBs containing ILVs can either fuse with the lysosomes or the plasma membrane, probably depending on their composition [23,24]. When MVBs are fused with the lysosome, lysosomal enzymes lead to degradation and the recycling of their content. Alternatively, when MVBs are fused with the plasma membrane, the carrying molecules might act as mediators in biochemical signaling in adjacent or remote recipient cells. Therefore, it is not yet elucidated how the fate of MVBs are determined. However, several recent studies suggest that the secretion of exosomes through fusion with the plasma membrane is regulated by Rab GTPase families such as Rab27 or Rab35 [25,26,27]. The fusion of MVBs with the plasma membrane is controlled by the soluble NSF-attachment protein receptor complex, which is also involved in the conventional calcium-regulated exocytosis of lysosomes [28].

### 1.2. Constituents of EVs: Proteins, miRNAs, Lipids and other Nucleic Acids

Molecular species and relative amounts in EVs are highly heterogeneous and complex in composition since they are loaded with a variety of molecular cargoes, such as nucleic acid, lipids and proteins, into both the surface and lumen of vesicles, which might be dependent on their cellular origin or microenvironment [29]. 

It has been widely accepted that EVs can provide a good source of biomarkers for the diagnosis or prediction of various diseases, such as cancer, metabolic diseases, cardiovascular diseases and neurodegenerative diseases; they also can be useful tools for drug delivery [30,31,32,33,34]. However, the nature and characteristics, as well as regulatory mechanisms of biogenesis, sorting and degradation or secretion of molecular contents in specific EVs (e.g., exosomes) are not fully understood. Analysis of the molecular contents of exosomes from distinct cell types or body fluids strongly suggested the context-dependent differentiation of concentrations of specific molecules in EVs [35,36]. EVs are enriched with lipids and proteins which are probably associated during membrane budding and share common features with the cell origin, such as glycosphingolipid, cholesterol, phosphatidylserine and ceramide [37,38]. Additionally, specific surface marker proteins are commonly found in exosomes, including tetraspanins (e.g., CD9, CD63, CD81), Alix, HSP70, GTPase and MHC molecules [39,40]. Apart from the membrane-associated proteins, exosomes isolated from cerebrospinal fluid (CSF) were also rich in proteins derived from the brain, such as microglial markers (CD11b and CD45), neuron-specific markers and apolipoprotein E (Apo-E), involved with neurodegenerative diseases [41]. Furthermore, a diverse composition of genetic materials (mRNA, microRNA and mitochondrial DNA) are also found in EVs. Among them, microRNA (miRNA) have attracted the most attention, due to their roles in the regulation of gene expression [42,43,44]. The mechanisms of sorting nucleic acids in exosomes are not fully understood. Nevertheless, many studies revealed that the exosomes derived from cancer cells contained similar miRNA content to their parent cells and, hence, exosomal miRNAs can be used as biomarkers [43,45,46,47]. Broad and accumulative studies of EVs have also opened up the possibility of using EVs as a source of biomarkers. Datasets, as described in the online resources comprising Exocarta, Vesiclepedia and EVpedia, have been gathered to identify the compositions of EVs from multiple organisms [48,49]. This is valuable information, which can help in the understanding and identification of active molecules involved in EV biogenesis and markers of disease progression in the pathological phase; for example, providing an early biomarker for neurodegenerative diseases. 

## 2. Physiological Role of EVs in Brain

EVs are considered as multifunctional molecular complexes which control the fundamental and homeostatic functions of cells. In the brain, EVs secrete various molecules associated with neuronal function and neurotransmission, thereby contributing to the reciprocal communication between neural cells (e.g., neuron-glia interaction), synaptic plasticity and neuronal activity [50,51,52]. In particular, the refinement and maintenance of synaptic connectivity in the adult brain are crucial in the cognitive function of the brain. The neural synaptic plasticity is regulated not only by neuron-specific processes but also by the contribution of glial cells, such as microglia and astrocytes [53]. In the regulation of synaptic plasticity, EVs from neurons can trigger synaptic pruning by microglia [54]. In addition, Frühbeis et al. have shown that exosome-like EVs participate in reciprocal oligodendrocyte-neuron communication and transfer cargoes from oligodendrocytes to neurons [55]. Furthermore, EVs might act as modulators to the physiological state of the recipient cells. For instance, miRNAs in exosomes isolated from a conditioned medium of neuronal culture (especially, miR-124a) regulate excitatory amino acid transporter 2, an essential mediator of glutamate uptake through the internalization of exosomes into astrocytes [56]. Moreover, it has been reported that exosomes released by cortical neurons upon activation of the glutamatergic synapse are selectively transferred to neurons [57]. 

Hence, EVs serve as vehicles for cell-to-cell communication in the brain by transferring molecules from diverse origins. Although exosome-mediated communication and its physiological significance in the CNS are largely unknown, there is emerging evidence to suggest that the shedding of neuronal EVs at the synapses could be functionally relevant for plasticity-associated processes [58]. For example, neuronal exosomes carry AMPA receptors, which might play a role in synaptic plasticity by regulating the number of AMPA receptors for glutamate transmission [59]. Furthermore, recent evidence points to the role of exosomes in regulating synaptic pruning. Exosomes released from PC12 cells facilitate synaptic elimination of microglia MG6 cells through increased complement component 3 expression [54]. In other studies, EVs from microglia and oligodendrocytes have also been proposed to support neuronal energy metabolism by carrying several enzymes involved in energy metabolism [60,61]. Thus, EVs mediate several vital processes involved in brain function.

While EVs mediate brain function and neuroprotection, there are several studies supporting the roles of EVs in the pathogenesis of neurodegenerative diseases. Exosomes contain a variety of proteins which are implicated in neural function and disorders, such as alpha-synuclein [62], tau [33], amyloid precursor protein (APP), APP C-terminal fragments and amyloid intracellular domain [63]. A study by Dinkins et al. showed that exosomes enhance the aggregation of amyloid-β 1-42 (Aβ1-42) [64]. In addition, it has been reported that exosomes from Alzheimer’s affected brains contain an elevated amount of Aβ oligomer and could act as a neuron-to-neuron vehicle for toxic species [65]. Moreover, Asai et al. have reported that microglia-derived exosomes lead to the progression of tauopathy [66]. Given the evidence, EVs may play a major role in neurodegenerative diseases and can be found in the plasma as well as in CSF, which allows the molecular cargoes of EVs to be used as biomarkers for neurologic diseases (explained in more detail below). 

## 3. Alzheimer’s Disease (AD), a Proteinopathy

AD is a major type of dementia, characterized by a group of symptoms associated with a decline in memory cognition and executive function which hampers daily life. Although AD is the most common neurodegenerative disease, its primary cause is poorly understood. In spite of some controversy, it is most widely accepted that the accumulation of Aβ and formation of intracellular neurofibrillary tangles composed of hyperphosphorylated tau aggregates leads to synaptic dysfunction, inflammation and neuronal loss [67,68]. Aβ is a short peptide derived from the sequential processing of APP by α-, β- and γ-secretases. When APP is cleaved by β- and γ-secretase, amyloidogenic Aβ fragments (Aβ1-42 and Aβ1-40) are produced. Aβ monomers are relatively non-toxic, while their oligomers are neurotoxic. The amyloidogenic Aβ peptides are mainly exported outside of the cells through exosomes, where the Aβ peptides produce neurotoxic amyloid oligomers, called amyloid plaques. Hyperphosphorylated tau, mediated by activation of tau kinases (e.g., glycogen synthase kinase-3b, cyclin-dependent kinase 5 (Cdk5) and protein kinase A) and/or inhibition of tau phosphatase (e.g., protein phosphatase 2A), generates neurofibrillary tangles inside neurons [69]. As Aβ plaques and tau tangles are generated by the aggregation of misfolded proteins, clearance of aggregated proteins is one of the therapeutic strategies against AD. 

Autophagy, one of primary proteolytic mechanisms, is considered to be a crucial regulator of the generation and clearance of Aβ [70]. APP processing occurs in autophagosomes, the autophagy-specific double-membrane structures [71]. Besides the degradation of Aβ peptides by Aβ-degrading enzymes including neprilysin and insulin-degrading enzyme [72], Aβ can accumulate in the autophagosomes of dystrophic neurites—the main constituents of the neuritic plaque of sporadic AD—and is released from neurons in an autophagy-dependent pathway [73]. Degradation of pathogenic tau is also dependent on autophagy [74]. Furthermore, genes essential for autophagy are reported to be implicated in AD pathology. Several studies reported that the expression of Beclin1, a main regulator of autophagy initiation, was reduced in AD patients [75,76,77]. In transgenic mice expressing human APP, a genetic reduction of Beclin1 leads to decrease in autophagy, accumulation of Aβ and neurodegeneration. Restoration of Beclin1 expression decreases the amyloid pathology in APP-transgenic mice [76]. Another critical regulator of autophagy, presenilin-1 (PSEN1) is also a main component of the γ-secretase complex. PSEN1 has been identified as a major genetic risk factor for early onset familial AD [78]. PSEN1 mutations impair its protein stability and AD patients bearing the mutations showed lower levels of Aβ in the CSF (i.e., reflected more accumulation of Aβ in the brain parenchyma) than AD patients without mutations [79,80]. During autophagy, PSEN1 regulates lysosomal acidification and proteolysis, which is consistent with the report of neurons in AD patients bearing PSEN1 mutation demonstrating lysosomal pathology [81,82]. Thus, it is believed that accumulation of Aβ in AD patients with PSEN1 mutations is caused by dysregulated proteolysis mediated by the autophagy-lysosome system.

Recent studies suggest that autophagy and exosome biogenesis are not only linked by the endolysosomal pathway but also share the same molecular machinery [83,84,85]. The early endosomes mature to multivesicular bodies (MVBs)/late endosomes containing intraluminal vesicles (ILVs). Exosomes are generated by the fusion of MVBs with the plasma membrane and the release of ILVs into the extracellular space. On the other hand, autophagosomes fuse with MVBs to generate amphisomes, which eventually fuse with lysosomes. More directly, ATG5, an essential gene for autophagy, can induce exosome release [86]. ATG5 inhibits the acidification of MVBs by disrupting V_1_V_0_-ATPase, which promotes exosome production independent of canonical autophagy. The ATG12-ATG3 complex, which is required for LC3 lipidation, interacts with Alix, the ESCRT-associated protein crucial for exosome biogenesis [87]. By interacting with Alix, the ATG12-ATG3 complex influences multiple Alix-mediated process including exosome biogenesis. As depletion of ATG7, another essential gene for autophagy, does not reduce exosome release, general autophagic function may not be required for exosome biogenesis [86].

In AD brains, Aβ plaques and tau tangles are often accompanied by other inclusions, such as transactive response DNA-binding protein 43 (TDP-43) or α-synuclein [88]. In fact, a significant number of sporadic AD patients have another pathologic comorbidities at autopsy, including Lewy bodies (LBs), TDP-43, vascular diseases or hippocampal sclerosis [89,90,91]. As these inclusions have also been identified in other neurodegenerative diseases, TDP-43 or α-synuclein cannot be considered a characteristic of AD [88,92]. However, TDP-43 can be shown the progression of AD, as patients with Aβ, tau and TDP-43 proteinopathy show more severe dementia than patients with Aβ or tau proteinopathy alone [93]. 

During the early phase, AD brains display a significant decrease in cerebral blood flow and a capillary amyloid angiopathy, both of which could augment the pathogenesis of AD [94]. Mechanistically, ischemia or hypoxia increases the activities of β- and γ-secretases and decreases the activity of α-secretase, resulting in the accumulation of amyloidogenic Aβ peptides in the brain. These changes are mainly mediated by hypoxia-inducible factor-1α (HIF-1α). HIF-1α increases the transcription of β-secretase (BACE1) through the hypoxia-response element and activates the γ-secretase complex through a direct interaction [95,96].

Currently, there is no method for confirmative diagnosis of AD in a live patient and neuropathologic findings in autopsied brains (i.e., amyloid plaque and neurofibrillary tangles) are the only confirmative methods in diagnosing AD. Several modalities have been developed during past decades and effort has been put forward to create diagnostic guidelines for “preclinical” and “clinical” AD (mild cognitive impairment (MCI) and AD) for research purposes by the National Institute on Aging and the Alzheimer’s Association in 2011 [97,98]. However, clinical diagnostic methods for AD are still largely dependent on the clinical assessment of neuropsychiatric function. To support the clinical diagnosis having low diagnostic sensitivity and specificity particularly in non-experts, positron-emission tomography (PET) imaging for amyloid plaques (AP) and tau tangles, magnetic resonance imaging (MRI) for the assessment of brain structure (e.g., hippocampal volume and cortical thickness), a 18F-fluorodeoxyglucose (FDG)-PET imaging for the assessment of brain glucose metabolism and the biochemical assessment of Aβ and tau in CSF can be applied [99,100,101,102]. Measurement of Aβ1-42, t-tau and p-tau181 in CSF using immunoassay platforms (e.g., ELISA or Luminex-xMAP) showed excellent diagnostic accuracy in various cohort studies, although the diagnostic cut-off values across the cohorts or immunoassay methods are variable [103]. 

## 4. Pathological Roles of Exosomes in AD

**Figure 1:**
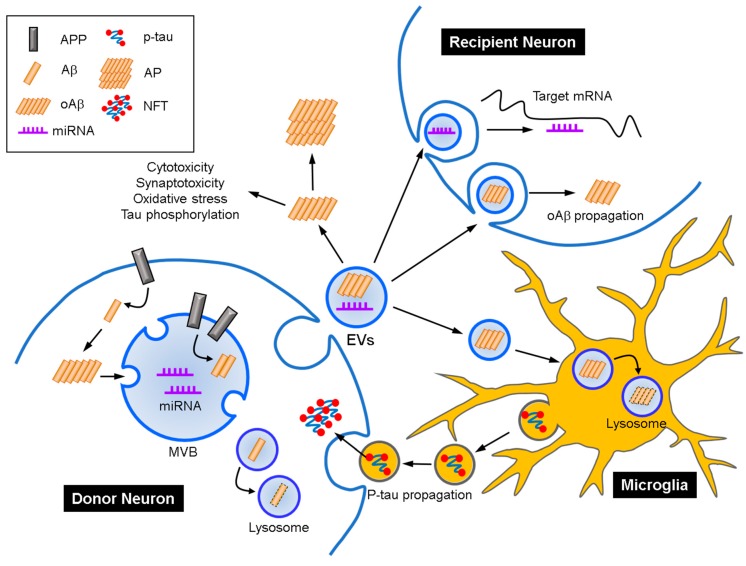
Potential pathological roles of extracellular vesicles (EVs) in Alzheimer’s disease (AD). Neurons release EVs containing Aβ monomers and oligomers (oAβ) and miRNAs. Microglia take in Aβ-associated EVs, then degrade Aβ using the lysosomal system. Exosome-associated Aβ can produce neurotoxic amyloid plaques (AP) outside the cells, when microglia-mediated clearance does not work properly. On the other hand, the EVs enter adjacent neurons by clathrin-mediated endocytosis or pinocytosis. Then, miRNAs promote the Aβ-generating pathway by targeting the mRNAs which are involved in amyloid precursor protein (APP) processing directly or indirectly. Conversely, hyperphosphorylated tau (p-tau) is transferred from microglia to neurons through EVs. Altogether, EVs may act as a main pathway in the propagation of protein aggregates between cells in the central nervous system. MVB, multivesicular body; NFT, neurofibrillary tangle.

Currently-marketed AD medicines seek to improve the clinical symptoms by targeting neurotransmitter neuronal circuits but not by modifying the underlying pathogenic mechanisms. For example, the pharmacodynamic targets of acetylcholinesterase inhibitors and NMDA receptor antagonists are not related to the pathogenic proteinopathies of AD. During the last decade, a series of drugs developed for inhibiting AD progression (i.e., disease-modifying therapeutics) tried to control Aβ aggregates but have all failed in the final stages of the clinical trial. It is still unclear how Aβ accumulation is triggered and the accumulated amyloidogenic Aβ and subsequent tau proteinopathy spread throughout the brain. Although the cellular and molecular mechanisms for the propagation of aberrant protein aggregates from the entorhinal cortex to midbrain a still not fully understood, compelling evidences suggest that propagation of protein aggregates via cell-to-cell transmission is one of the mechanisms of AD progression [104]. The concept of spreading of disease by protein aggregates in the CNS confined to prions has now expanded to many neurological disorders including AD [105]. In an animal model, exogenous inoculation of Aβ-containing brain extracts spreads Aβ at the injected site, as well as in the adjacent regions [106,107]. In addition, the inhibition of exosomal secretory pathways and the pharmacological inhibition of exosome synthesis in microglia halts tau propagation and amyloid plaque load in vitro and in vivo [66,108]. Furthermore, Aβ and tau have been detected in exosomes secreted into the extracellular space [109]. These studies suggest that exosomes may be the main route governing the propagation of Aβ and tau (Figure 1).

Alix and flotillin-1, the marker proteins in exosome-like vesicles, are enriched in the amyloid plaques of AD patient brains [109]. In addition, Aβ can bind to GM1 ganglioside, which is abundant in exosomes, suggesting that GM1 ganglioside serves as an attachment site for Aβ at the exosomes [110]. GM1 ganglioside-bound Aβ has a distinct structure from soluble Aβ and initiates Aβ aggregation by acting as a seed [110]. Neuron-derived exosomes can also accelerate Aβ fibril formation [108,111]. The exosome-associated Aβ is actively taken into microglia, brain-resident macrophages, where the Aβ is degraded by the lysosomal system. Meanwhile, administration of Aβ-trapped exosomes into AD mouse brains reduces Aβ pathology [112]. Thus, exosomes are thought to support the propagation and clearance of Aβ, providing a novel therapeutic approach for AD. However, it should be noted that the exosome-mediated approach could only be effective when the microglia are functioning normally.

An increase in protein levels of total and phosphorylated tau in exosomes has been observed in the CSF at an early phase of AD [33,66]. Indeed, tau is transmitted from microglia to neurons via exosomes [66]. Depletion of microglia or inhibition of exosomes suppresses tau propagation. In contrast to Aβ, the mechanism by which cytosolic tau is assembled into exosomes has not been solved yet. 

As described earlier, miRNAs are important components of exosomes, which are transferred between cells via exosomes and affect the behavior of recipient cells. Numerous studies have shown that the levels of several kinds of miRNAs are altered in exosomes from various sources (brain, blood and CSF) of AD patients [113,114]. Interestingly, most of the miRNAs are reduced in AD patients, while small number of miRNAs are increased. Among those, many miRNAs are identified to be involved in AD pathogenesis. Some miRNAs, including miR-29a, -29b-1, -107 and -195, regulate the expression of BACE1/β-secretase, whose activity is the rate-limiting step in Aβ production [115,116,117]. In the brain of AD patients, the miR-29a/b-1 cluster is significantly decreased and protein levels of BACE1 are increased [115]. Both miR-29a and -29b-1 bind to the 3′-UTR of BACE1 in vitro and regulate the protein expression of BACE1. The protein levels of BACE1 in AD patients are negatively correlated with expression levels of both miRNAs. Additionally, miR-107 and -195 show negative correlations with the BACE1 expression levels [116,117]. Other miRNAs regulate the expression of proteins involved in the APP processing pathway, such as APP and ADAM10/α-secretase [118,119,120]. These studies suggest that many miRNAs are involved in AD pathogenesis through the APP processing pathway. In addition, there is evidence that miRNAs act in AD pathogenesis by regulating tau. In AD patients, a decrease in the miR-132-3p level is accompanied with hyper-phosphorylation of tau [121]. It appears that miR-132-3p targets the Forkhead (FOX) transcription factor, FOXO1a, to regulate tau. Additionally, miR-125b, which is increased in AD patients, can promote tau phosphorylation by targeting FOXQ1, another FOX transcription factor [122]. And miR-26b, which is also increased in AD patients, induces nuclear export and activation of Cdk5, a neuronal kinase involved in the phosphorylation of tau [123].

## 5. Biochemical Biomarkers for Early Diagnosis of Alzheimer’s Disease

A molecular biomarker is defined as a molecular characteristic that is objectively measured and evaluated as an indicator of a normal physiology, pathologic process or pharmacological response to a therapeutic intervention [124]. Aβ and hyperphosphorylated tau protein are the molecules responsible for the pathological hallmarks of AD (i.e., amyloid plaque and neurofibrillary tangle, respectively). CSF is the best biofluid that reflects molecular events in the brain and hence molecules in CSF are the first candidates for diagnostic biomarkers for AD. Until now, measurements of Aβ1-42, total tau and p-tau181 in CSF are the best molecular markers for the early diagnosis of AD, although the relative invasiveness of CSF collection over drawing blood is a major limit in measuring CSF AD biomarkers [125,126,127]. Currently, enormous efforts in developing a valid biomarker in blood are being made and various molecular candidates for early diagnosis of AD have been proposed. Among the proposed biomarkers, molecules extracted from EVs, particularly from exosome-like EVs have shown good diagnostic accuracy. Nevertheless, definitive evidence for the clinical validity of EV-derived molecules in CSF and blood remains elusive. 

### 5.1. Non-Amyloid Protein Biomarkers in CSF 

Biomarkers can be useful in optimizing therapeutic clinical trials and providing the pharmacodynamic efficacy or target engagement of a developed disease-modifying drug. In addition, biomarkers can differentiate the heterogeneity of AD (e.g., severity, heterogeneous progression or mixed non-amyloid pathology). Several non-amyloid biomarkers in CSF have been suggested to be complementary to amyloid or tau pathology. The levels of neurogranin in CSF of AD or mild cognitive impairment (MCI) patients were higher than in cognitively-normal elderly patients [128,129]. Neurogranin is a marker for synaptic integrity, which is concentrated in the dendritic spines of excitatory synapses and plays a role in long-term potentiation [130]. Synaptic loss, as a fundamental and early pathophysiological mechanism of AD, may be associated with a higher level of neurogranin in CSF of MCI or AD patients than in the control group. Furthermore, a higher level of CSF neurogranin was associated with the rapid progression of AD, particularly in patients with Aβ pathology [131]. More importantly, a higher level of CSF neurogranin was not observed in non-AD neurodegenerative diseases [132,133], indicating that neurogranin may be specific to AD. Neurofilament light (NFL) is another non-amyloid biomarker candidate in the CSF. NFL is an axonal protein and, therefore, it reflects axonal damage in white matter. A higher level of NFL in CSF can be a marker of amyloid-independent neurodegeneration in MCI or AD. However, the level of NFL in CSF of other neurodegenerative diseases (e.g., frontotemporal dementia) was also higher than in the control group [134]. Taking these non-amyloid CSF biomarkers together with ‘core AD biomarkers’ (Aβ1-42, total tau and p-tau181) further increased the diagnostic accuracy of the core AD biomarkers [135]. In addition, the emerging non-amyloid CSF biomarkers can be useful to discriminate AD from non-AD (e.g., dementia with Lewy bodies), predict the AD progression or monitor the pharmacodynamics in a treatment trial. 

### 5.2. Protein Biomarkers in Plasma and Circulating EVs

In the blood, the extremely low concentration of brain-derived molecules and a complicated matrix hamper the development of blood AD biomarkers and requires technological improvements in analytical accuracy to measure the circulating biomarkers. For example, the measurement of Aβ species in plasma using a conventional ELISA method showed conflicting results [136,137]. The conflicting results may result from the analytical performance of the immunoassay platform, which is influenced by an abundance of plasma proteins (i.e., albumin, autoantibodies and heterophilic antibodies) and the higher complexity of the matrix when compared to CSF (e.g., high concentration of lipids). To overcome the limitation of conventional immunoassay platforms in measuring Aβ in blood, novel analytical technologies have been developed. For example, immunomagnetic reduction assays [138] or single molecule arrays (SIMOA) have been applied to detect Aβ or tau in plasma [139,140]. Immunoprecipitation (IP)-coupled mass spectrometry was used to measure Aβ species (APP(699-711), Aβ1-42 and Aβ1-40) in the blood with a good predictability of amyloid positivity in a large cohort [141]. However, the overall results for plasma Aβ1-42 concentration measured by novel technologies still showed inconsistent results (meta-analysis results can be found in; http://www.alzforum.org/alzbiomarker/meta-analysis/alzheimers-disease-vs-control-av42-plasma-and-serum, ver.2.0, 2017). Recently, neural-derived proteins in exosomes have been proposed as blood AD biomarkers. The neural-derived exosomes, isolated from plasma of AD patients, showed significantly higher levels of Aβ1-42, total tau, p-T181 tau and p-S396 tau, as compared to the controls, which provided a high predictability of disease development in the preclinical stage [142]. In diabetic patients, the level of the phosphorylated form of insulin receptor substrate 1 in neural-derived exosomes also showed a higher accuracy in predicting the development of AD [143], which is associated with regional brain atrophy [144]. The increased levels of lysosomal proteins (i.e., cathepsin D and LAMP1) and decreased levels of synaptic proteins (synaptophysin, synaptopodin, synaptotagmin-2 and neurogranin) were also observed in the neuron-derived plasma exosomes of AD patients [145,146]. Goetzl and colleagues found that the levels of cargo proteins in plasma exosomes derived from astrocytes were higher than those in neuron-derived plasma exosomes, which implicates that plasma exosomal biomarkers from different neural cells may be useful in investigating the mechanisms of cellular interactions and the effects of AD therapeutics [147]. 

### 5.3. microRNA in EVs of Plasma and CSF

In addition to proteins, a good molecular context for blood AD biomarkers is miRNA. miRNA is a small (22–23 nucleotides) non-coding RNA which suppresses translation or induces degradation of multiple target mRNA by binding to the 3′-untranslated region. miRNA is very stable in biofluids and can be attributed to the pathogenesis of a specific disease. miRNA can be released into extracellular fluid and circulated by binding to RNA-binding proteins (e.g., AGO-2 or high-density lipoprotein) or incorporation into the EVs. In experimental models of AD or clinical studies, various miRNAs were suggested to be involved in AD pathogenesis. Deregulated expression of miRNAs may contribute to the regulation of key genes involved in AD, including amyloid production [116,117,148,149,150] and tau regulation [121,123,151], although it remains to be elucidated whether the correlation between the levels of miRNA expression and AD pathology is a cause or a consequence of the disease. Determination of the miRNA profile in CSF of AD may be important in understanding the AD heterogeneity or to discover therapeutic targets for AD. However, if the blood is contaminated during CSF collection, it will be an important confounding factor when performing miRNA detection in CSF. In a multicenter validation study [152], it was reported that the measurement of miRNA in CSF can be biased by several pre-analytical and analytical sources of variability, which may produce inconsistent results. Previous studies using CSF from a relatively small number of subjects showed that several miRNAs were up-regulated or down-regulated in CSF of AD patients; however, the results were not consistent [152,153,154,155,156,157]. Therefore, the identification of confounding factors when analyzing miRNA in CSF is required. 

Using blood samples, numerous studies reported the diagnostic utility of various miRNAs in plasma, serum, whole blood or peripheral blood mononuclear cells (PBMCs). Single miRNA or a panel of miRNAs showed diagnostic sensitivity and specificity ranging from 68 to 100% in clinical studies with a large (N > 100) number of subjects (see Table 1). It should be noted that not all miRNAs showed consistent results. For instance, miR-29b, miR-181c, miR-15b, miR-146a and miR-107 were consistently downregulated in AD blood; however, miR-34a, miR-143, miR-26b, miR-let-7f, miR-138, miR-135a, miR-200b and miR-93 showed inconsistent results. It is not clear why some results are inconsistent. Several factors can be considered; First, the standardization and validation of the extraction procedure of RNA for detection of miRNA may be poor. As the concentration of circulating miRNA is very low, the library construction to determine the profile of scant miRNA species may not be stable. Second, pre-analytical factors, such as clinical heterogeneity or severity, can be a cause of inconsistent results. In fact, it has been reported that the miRNA profile between MCI and AD is different [158]. Other clinical biases (diet, drugs or comorbidity) can be biasing factors. Third, the method of analysis for miRNA quantification is variable (i.e., next-generation sequencing (NGS) versus quantitative real time PCR (qRT-PCR)). Finally, the preparation method for the separation of plasma, serum or PBMC or for use of whole blood or extraction of EVs is critical in observing consistency. In particular, the standardization of EV extraction from blood constituents (plasma or serum) for EV-miRNA biomarker development is more complicated. Conceptually, miRNAs reflecting the brain pathology in AD are significantly less than the miRNAs from other peripheral tissues. A recent study suggested that the yield of EV-associated RNA is less than 5 ng per mL of biofluid [159], which is four- to ten- fold lower than RNA from total plasma (20–50 ng/mL). The low amount of RNA for subsequent NGS library construction or qRT-PCR can lead to an unintentional bias (e.g., production of adaptor dimer by-products). Recently, a report outlining biofluid collection and preparation for EV-miRNA analysis by members of the Extracellular RNA Communication Consortium elegantly reviewed the current methodologies and found both unique challenges and unprecedented opportunities [160]. In addition to the bottleneck of low concentration of circulating EV-miRNA, determination of endogenous control miRNA for qRT-PCR normalization in blood analysis should be considered [161].

## 6. Consideration to Develop AD Biomarkers in EVs

EVs (e.g., exosomes) were discovered over decades ago and were considered to play a role in cellular garbage disposal. However, more recent studies have strongly suggested that EVs play roles in intercellular communication. Nevertheless, it should be noted that EV subtypes (i.e., endosome-origin exosomes and plasma membrane-derived microvesicles) cannot be easily defined, due to a lack of consensus on specific markers for the subtypes. Therefore, unless specific markers of subcellular origin which are reliable within experimental system(s) are not established, we should consider the use of operational terms for EV subtypes, such as small EVs. In this review, we used conventional terms for EV subtypes, following previous reports; however, we do not recommend the use of nomenclature as such ‘exosome’ without clear evidence of the subcellular origin. 

One of the highlights of EV research is discovering the molecular characteristics specific to certain diseases. In the field of AD biomarker research, considerable attention has been drawn to EV-derived biomarkers, particularly in blood. However, several issues for valid EV AD biomarker development remains to be elucidated. 

### 6.1. Standardization of Pre-Analytical Sources of Variability for EV AD Biomarkers

During the development of CSF ‘core biomarkers’ for the early diagnosis of AD, particularly in CSF Aβ1-42, pre-analytical sources of variability are one of the most important issues in quantifying the level of Aβ1-42 in CSF. For instance, the material of the tube for CSF collection and storage is one of the pre-analytical sources and it has been widely accepted that a polypropylene tube should be used for CSF Aβ1-42 quantification. Until now, there has been limited consensus on the standardization of pre-analytical sources of variability, including (but not limited to) demographics of patients, diet before sampling, diurnal variation, sample collection and preparation procedure, sample storage condition and use of anticoagulant for blood EV isolation. All steps from sample collection to sample storage (e.g., blood collection, centrifugation, aliquot and storage in freezer) should be determined according to the type of body fluid. As the characteristics of body fluids (e.g., viscosity or protein concentration) or sample preparation procedures (e.g., temperature, centrifugation or use of an anti-coagulant) may affect the yield and/or size distribution of EVs [176,177], the standardization of these sources of variability is necessary for the development of valid EV biomarkers. In addition, clinical factors, as a source of pre-analytical variability, should be considered. The effects of comorbidity, use of specific drugs and diet condition before sampling on the yield of EV isolation should be determined. 

### 6.2. Yield and Purity of Isolated EVs from Biofluid

Several methodologies capable of isolating EVs from body fluids have been introduced. Since there are pros and cons for each methodology in terms of preparation, no reference method for EV purification has been established. Furthermore, the sizes and densities of the vesicles overlap between EV subtypes and, hence, it is hard to clearly distinguish between the subtypes by size-exclusion chromatography, differential ultracentrifugation, precipitation or density-gradient separation. Isolation of pure exosomes from samples in vitro is generally performed by a cushioned density-gradient centrifugation (e.g., a concentrated cell culture medium); however, the method is unlikely oriented to clinical practice due to the low yield. Differential ultracentrifugation or a commercial kit using precipitation methods increases the yield of exosomes; however, the contamination of other extracellular molecules (e.g., lipoproteins) in biofluids may significantly interfere with the biomarker development. In blood AD biomarker studies using ‘neural exosomes’ prepared by exosome precipitation and neuron- or astrocyte-specific antibody capture, several biomarkers have been suggested as blood AD biomarker with high diagnostic accuracy [142,143,146,147]. However, it should be further elucidated whether the characteristics and purity of ‘neural exosomes’ from very small volumes of plasma or serum can clearly define ‘exosomes,’ as described above. In particular, ‘exosomes’ prepared from plasma by commercial kits using a precipitation method showed high contaminations of non-exosomal molecules [178,179]. Therefore, the purity of exosomes isolated by a commercial precipitation kit should be carefully tested. In addition, the biochemical and physical properties of body fluids are diverse and the optimal isolation method for each body fluid should be established to minimize the contamination of non-vesicular materials or aggregation of exosomes during preparation. For instance, serum or plasma may have different yields of EVs. Platelet-derived EVs can be released after blood collection during the process of clot formation [180]; hence, the yield of EV in serum may be higher than that in plasma [181]. In contrast, the natural physiological medium of EV in the blood is plasma. Therefore, additional studies are required to increase our understanding of plasma/serum differences and how they are influenced by sample processing procedures. For the use of anticoagulants during blood collection, trisodium salt of citrate or acid citrate dextrose may be superior to other anticoagulants in EV research, although further studies are necessary [182,183]. 

Both qualitative and quantitative approaches, including morphological observation using TEM, western blotting of exosomal marker proteins, size distribution and number of vesicles by dynamic light scattering or nanoparticle tracking analysis, can be applied to estimate the purity of exosomes. To define the purity of EVs, a consensus on the ‘minimal information studies of EV’ based on a discussion by the Executive Committee of the International Society for Extracellular Vesicles has been published [184,185]. They proposed several criteria, which represent the minimal characterization of EVs when reported by investigators. For RNA research using EVs, several papers were also published to define the purity of prepared vesicles using various isolation methods [160,186,187]. The minimal requirements to claim presence of EVs in isolates are as follows: (1) EVs are isolated from extracellular fluids-that is, from conditioned cell culture mediums or body fluids; (2) EVs are defined by quantitative measures of the source of EVs (e.g., number of secreting cells and volume of biofluid); (3) Characterization of vesicles using at least two different technologies should be performed; (4) Preparation of EVs should be characterized to the extent possible to determine abundance of EVs (particle number and protein content); and (5) Purity of EVs should be determined by testing for the presence of components associated with EV subtypes, depending on the specificity one wishes to achieve, as well as for the absence of non-vesicular components. More importantly, detailed experimental protocols to isolate EVs from the body fluid, as well as their characteristics, should be presented in a publication [185].

In a clinical setting, the use of large sample volume of biofluid may not be appropriate for diagnostic purposes and, therefore, the isolation of EVs with high purity and yield using a small volume of biofluid is critical to the clinical application of disease biomarkers. Therefore, methods having an ability to isolate EVs with higher purities and yields should be developed for EV biomarker development. For instance, the removal of non-vesicular proteins with high-molecular weight or protein aggregates which can be co-sedimented with small EVs during ultracentrifugation is important in analyzing EV biomarkers. Non-exosomal miRNA binding to ribonucleoprotein Aog2 can be a bias of exosomal miRNA biomarker discovery [188], rendering Ago2 as a potential reliable marker to evaluate the contamination of exosome preparation for miRNA biomarker research. 

Given a low quantity, the application of analytical methods with a high sensitivity for the quantification of EV proteins from a small volume of biofluid might be necessary. In particular, mass spectrometry (MS)-based proteomic analysis for the discovery of EV protein biomarkers has boosted the knowledge of disease-specific protein context in EVs [189]. Furthermore, the application of acquisition methods such as selected reaction monitoring or parallel reaction monitoring with a MS instrument for selected peptides derived from parent protein of EV can obtain a higher analytical sensitivity [190]. In addition, liquid chromatography-MS (LC-MS) can be applied to evaluate the post-translational modification of EV proteins. For EV miRNA research, the low yield of EV miRNA can be a substantial bias during miRNA signature analysis, particularly during NGS library construction. In addition, the endogenous control for qRT-PCR normalization of target miRNA in EVs should be carefully determined, since no universal endogenous miRNA controls exist until now. A study reported that endogenous control for EV miRNA qRT-PCR analysis should be differentially applied according to the type of body fluid [161]. According to the International Society of Extracellular Vesicle (ISEV) survey at the end of 2015, the most commonly used EV isolation method is differential ultracentrifugation combined with other techniques. More recently, however, additional techniques or combinations of methods have been introduced [185] to improve the purity and yield of EVs, which can accelerate EV biomarker research and development. 

## 7. Future Directions

To develop valid EV biomarkers for the early diagnosis of AD (or prediction of the disease), we are faced with several challenges. First, the identification of various sources, awareness of potential pitfalls, determination of EV isolation efficiency in various biofluids, standardization of EV preparation (including sample collection, preparation, storage and EV isolation procedures) and improvement of EV purity and yield will be necessary. Second, we should consider the optimal methods for EV biomarker discovery and evaluate the reproducibility of AD signatures in independent cohorts. Third, during the clinical validation processes, AD signatures in EV biomarkers should be evaluated, as to whether the signature is correlated with other valid non-EV AD biomarkers, such as amyloid PET positivity, CSF core AD biomarkers and clinical measures in large cohorts. Finally, for the clinical application of EV biomarkers, valid methods for the quantification of EV biomarkers and evidence of target engagement in a clinical trial are necessary (Table 2). Therefore, the development of valid EV biomarkers will take a long time and need enormous efforts. The efforts of the world-wide Alzheimer’s Disease Neuroimaging Initiative (ADNI) will be a benchmark for the development of EV AD biomarkers. The ADNI studies are supported by the Alzheimer’s Association (AA) and private industries and, hence, the development of EV biomarkers using ADNI samples and ADNI-independent samples will be an efficient way to facilitate the development of EV AD biomarkers. In addition, the International Society of Extracellular Vesicles may play a role in this field, in collaboration with AA.

## Figures and Tables

**Table 1:** Selected studies * using body fluids as a source of miRNA AD biomarkers.

Body Fluid	Study Groups	miRNA Analysis Method	miRNA	Sensitivity	Specificity	Reference
Whole blood	NC^#^ (21) MCI (18) AD (94)	qRT-PCR of miRNA discovered by NGS from 22 HC and 48 AD	12 miRNAs ^1^	95.1	91.5	[162]
Serum	NC (150) AD (105)	qRT-PCR	miR-125b	81	68	[163]
Serum	NC (50, 155) AD (50, 158)	qRT-PCR following Illumina sequencing	miR-342-3p 6 miRNAs ^2^	85 81	71 68	[164]
Serum	NC (48, 75) AD (48, 79)	qRT-PCR following Solexa sequencing	4 miRNAs (miR-31, miR-93, miR-143, miR-146a)	n.a.^$^ (AUC = 71 – 75)		[165]
Serum	MCI-MCI (330) MCI-AD (128)	qRT-PCR	miR-206	95.3	77.8	[166]
Serum	NC (76) MCI (66)	qRT-PCR	miR-206 miR-132 Combined	86.4 69.7 85.5	76.3 100 98.5	[167]
Serum	NC (62) AD (84)	qRT-PCR	miR-29 + miR-223 miR-125b + miR-223	n.a. (AUC = 0.826) n.a. (AUC = 0.879)		[168]
Plasma	NC (81) MCI (116) AD (97)	qRT-PCR	miR-107	98.3	82.7	[169]
Plasma	NC (50) MCI (50)	qRT-PCR	miR-132 family (3 pairs) miR-134 family (3 pairs)	84–94 (96) 76–88(80)	96–98 (96) 80–90(94)	[170]
Plasma	NC (85) AD (78)	qRT-PCR	miR-34c miR-34a	92 84	96 74	[171]
Exosome (serum)	NC (228) MCI (101) AD (107)	qRT-PCR	miR-135a miR-193b miR-384 Combined	90 78 85 99	95 77 90 95	[172]
Exosome (serum)	NC (36) MCI (8) AD (16)	qRT-PCR following discovery by sequencing from 23 HC, 3 MCI and 23 AD	16 miRNAs ^3^	87	77	[173]
Exosome (plasma)	NC (35) AD (35)	Illumina deep sequencing	Panel of 7 miRNAs ^4^	n.a. (AD prediction rate = 89%)		[174]
Exosome (plasma, CSF)	NC (7) MCI (43) AD (51)	qRT-PCR	miR-193b	n.a.		[119]
CSF	NC (40) MCI (37) AD (57)	qRT-PCR	No tested miRNA deregulated in AD (miR-29a, miR-125b-5p, miR-146a-5p, miR-16-5p, miR-24-3p)	n.a.	n.a.	[152]
PBMC	NC (344) AD (287)	qRT-PCR	miR-590-3p	n.a.	n.a.	[175]

* Studies with large number of subjects (N > 100) have been included, except for studies using exosomes. ^$^ Not available. ^1^ let-7f-5p, miR-1285-5p, miR-107, mir-103a, miR-26b-5p, miR-26a-5p, miR-532-5p, miR-151a-3p, miR-161, let-7d-3p, miR-112, miR-5010-3p; ^2^ miR-95-5p, miR-885-5p, miR-483-3p, miR-342-3p, miR-191-5p, let-7d-5p; ^3^ miR-361-5p, miR-30e-5p, miR-93-5p, miR-15a-5p, miR-143-3p, miR-335-5p, miR-106b-5p, miR-101–3p, miR-425-5p, miR-106a-5p, miR-18b-5p, miR-3065-5p, miR-20a-5p, miR-582-5p, miR-1306-5p, miR-342-3p, miR-15b-3p; ^4^ miR-342-3p, miR-141-3p, miR-342-5p, miR-23b-3p, miR-24-3p, miR-125b-5p, miR-152-3p. ^#^ Abbreviations: NC, normal control; MCI, mild cognitive impairment; AD, Alzheimer’s disease; NGS, next-generation sequencing; CSF, cerebrospinal fluid; PBMC, peripheral blood mononuclear cell; qRT-PCR, quantitative real-time polymerase chain reaction; miRNA, microRNA; AUC, area under the curve.

**Table 2:** Current challenges against the development of EV AD biomarkers and strategies to overcome the challenges.

Developmental Process	Current or Future Challenges	Required Strategies
1. EV preparation	- Low level of knowledge for the preclinical sources of variability - No standard EV preparation method to meet both high purity and yield - Deficiency in awareness of potential pitfalls	- Identification and standardization of the sources (e.g., sample collection, processing and storage and effects of diet or medicine on EV preparation) - Improvement and standardization of current methods or development of novel method for preparing EV with high purity and yield - Identification of potential pitfalls in each EV preparation method
2. Biomarker discovery	- Low level of knowledge for the analytical and clinical validity of EV AD biomarker candidates - Potential systemic bias during biomarker discovery (e.g., EV RNA extraction, NGS library construction)	- Evaluation of reproducibility of AD signatures from prepared EV and discovery of EV biomarker candidates in autopsy-confirmed AD cases - Improvement and standardization of current methods or development of novel discovery methods
3. Clinical validation	- Current need for large cohort studies to evaluate the AD diagnostic utility of specific EV biomarkers - Current need for clinical studies evaluating the correlation of EV biomarkers with well-known AD biomarkers	- Evaluation of AD diagnostic performance in multi-center studies in collaboration with academia and stakeholders - Evaluation of the correlation of EV biomarkers with neuropsychiatric testing and AD biomarkers including amyloid-PET or CSF core AD biomarkers
4. Clinical application	- Future need of feasible methodologies for EV biomarker quantification - Consumption of enormous resources to develop EV biomarkers	- Development of valid biomarker quantification methods and evidence that fulfils the clinical utility - Collaboration among academia, industry and regulatory agencies with the support of AA and ISEV to maximize cost-effectiveness
